# Passive catheter tracking with a controllable susceptibility effect

**DOI:** 10.1186/1532-429X-15-S1-P17

**Published:** 2013-01-30

**Authors:** William Dominguez-Viqueira, Hirad Karimi, Charles H Cunningham

**Affiliations:** 1 Imaging Research, Sunnybrook Research Institute, Toronto, ON, Canada; 2 Medical Biophysics, University of Toronto, Toronto, ON, Canada

## Background

Due to the rich anatomic information available, MRI is an attractive tool for guiding endovascular interventions. Susceptibility artifact-based tracking using paramagnetic markers [1] is a simple and economical approach, but has been used with limited enthusiasm partly because of the image degradation that results from such devices. In this work, a susceptibility-based tracking catheter which can be mechanically turned ON and OFF [2] is presented with images in phantoms and animals studies.

## Methods

**Figure 1:**
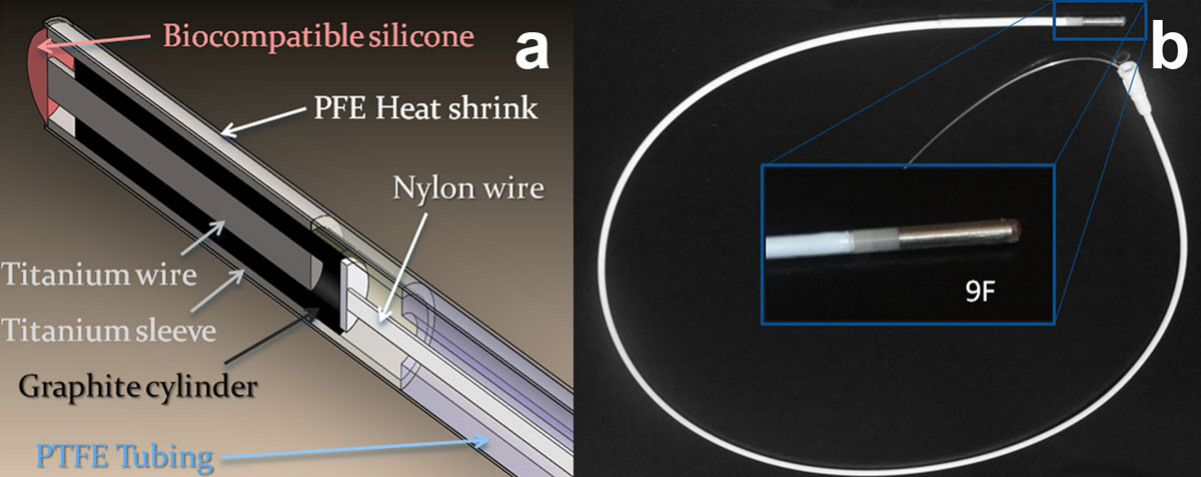
Catheter assembly with the susceptibility device. a: 3D model of the assembly. b: actual catheter picture.

The susceptibility device consisted of three concentric cylinders of titanium and graphite giving an outer diameter of 3 mm and length of 15 mm (Figure 1a). The device was designed to create a minimum susceptibility artifact in MRI when all the cylinders are aligned (OFF position); and a large artifact when the cylinders are miss-aligned (ON position) to facilitate tracking [2]. The catheter was built attaching the device to biocompatible PTFE tubing with heat shrink, gluing the titanium parts at the distal end. A nylon wire was glued the graphite piece on the opposite end of the device to push and pull this piece in and out relative to the titanium parts (Figure 1a and b). All the imaging experiments were performed using a 3T MR scanner (MR750, GE Healthcare, Waukesha, WI), with a 5 inch diameter receive-only surface coil using a fast gradient-recalled echo sequence. Volume projection images with positive contrast were acquired using a frequency selective spin echo sequence to find and track the catheter. The catheter was placed inside a doped-water bath (saline and copper sulfate) for phantoms experiments. In vivo studies were performed in a specific pathogen free (SPF) pig (25 kg) under a protocol approved by the institutional animal care and use committee.

## Results

**Figure 2:**
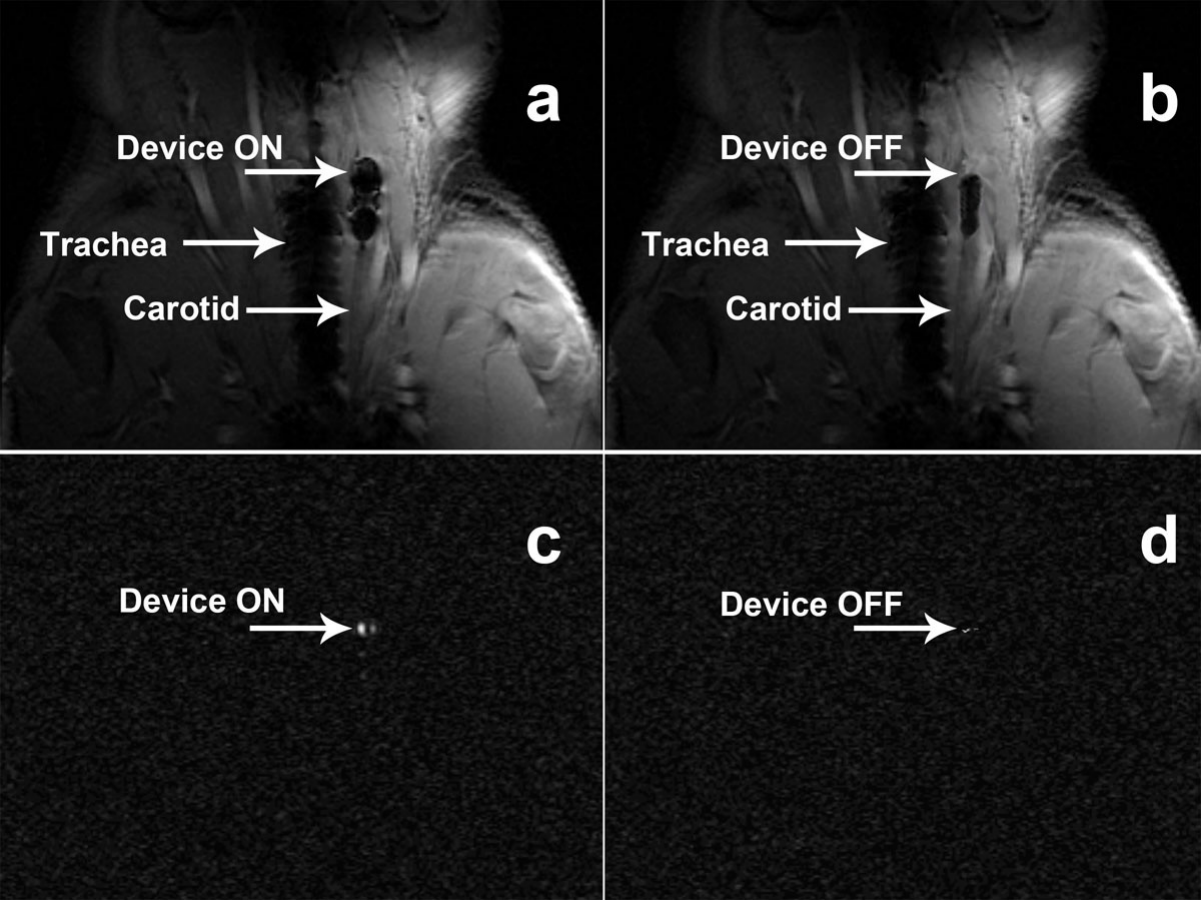
Images of the catheter *in vivo* acquired at 3T. a and b: fast GRE images of the neck area of a pig with the device in ON and OFF position respectively, c and d: positive contrast images of the same area with the device in ON and OFF position respectively.

In vivo images of the neck area of a pig with the catheter in ON(a) and OFF(b) positions are shown in Figure 2. As shown in Figure 2a and b the image distortions are minimized when the device is in the OFF position (Figure 2b). Positive contrast images are shown in Figure 2c and d, where only the tip of the device can be seen. This facilitates locating the device in projection images which can be helpful *in vivo* to automatically track the device.

## Conclusions

A passive tracking catheter with a susceptibility effect that can be enabled and disabled by sliding one of the components was designed, fabricated and demonstrated in vivo. The difference between the aligned and miss-aligned configurations was large in the acquired MR images, showing the feasibility of tracking the device by periodically moving the graphite layer. Even though the device was demonstrated in a catheter, it can also be designed for different tools or devices for interventional MR procedures. In future work, faster imaging sequences will be implemented for real-time tracking.

## Funding

Funding from the Canadian Institutes of Health Research (CIHR PPP106789).
